# DichroMatch at the protein circular dichroism data bank (DM@PCDDB): A web‐based tool for identifying protein nearest neighbors using circular dichroism spectroscopy

**DOI:** 10.1002/pro.3207

**Published:** 2017-10-25

**Authors:** Lee Whitmore, Lazaros Mavridis, B.A. Wallace, Robert W. Janes

**Affiliations:** ^1^ Institute of Structural and Molecular Biology, Birkbeck College, University of London London United Kingdom; ^2^ School of Biological and Chemical Sciences Queen Mary University of London London United Kingdom

**Keywords:** tools for protein characterization, bioinformatics, circular dichroism spectroscopy, secondary structure, folds

## Abstract

Circular dichroism spectroscopy is a well‐used, but simple method in structural biology for providing information on the secondary structure and folds of proteins. DichroMatch (DM@PCDDB) is an online tool that is newly available in the Protein Circular Dichroism Data Bank (PCDDB), which takes advantage of the wealth of spectral and metadata deposited therein, to enable identification of spectral nearest neighbors of a query protein based on four different methods of spectral matching. DM@PCDDB can potentially provide novel information about structural relationships between proteins and can be used in comparison studies of protein homologs and orthologs.

## Introduction

Circular Dichroism (CD) spectroscopy is a biophysical method that can be rapidly and facilely used to characterize protein structures in solution. It enables determination of protein secondary structures,^1^ and comparisons between spectra can be used to identify similarities and differences between proteins related by sequence or structural homologies. It is also useful for comparisons of the structures of the same protein under different conditions.

DichroMatch is a tool that was originally developed^2^ for identifying near‐neighbor protein CD spectra using either (1) a set of user‐provided spectra (as a means of comparing, for example, the structures of closely related proteins, different mutants of a protein or the same protein under different conditions), or (2) a compilation of the SP175^3^ and SMP180^4^ reference dataset spectra of soluble and membrane proteins, respectively, which have known crystal structures (as a means of identifying proteins with similar secondary structures and folds).

The DichroMatch functionality has now been incorporated within the Protein Circular Dichroism Data Bank^5^ (PCDDB) as DM@PCDDB, enabling matching with any spectra available in that data bank, including the components of the SP175 and SMP180 reference data sets, augmented with the other entries available in the PCDDB. At present there are more than 500 entries in the data base available for matching. Thus, this new version broadens, facilitates, and enhances the searchable range of protein spectra. Furthermore, as the number of entries in the data bank increase (through user depositions), the number of close proximity targets will increase and expand the range of protein structures that can successfully be queried.

DM@PCDDB thus provides a user‐friendly method for identifying structurally‐related proteins based on their CD spectra (even for proteins which do not have similar sequences).

## Matching Methods

The four matching methods in DM@PCDDB are as described in detail in Klose et al,^2^ and include: **Simple Fit,** which as its name suggests, does a match between the (unmodified) query and test spectra over the wavelength range specified; **Normalized Comparison**, which scales the test spectrum to the query spectra at their maxima, thereby eliminating overall spectral magnitude as a factor; **Ratio Comparison**, which compares only the ratios of the peaks at 195 nm, 208 nm, and 222 nm, often indicative of similar secondary structures, regardless of fold features; and **Wavelength Shift**, which enables the shift of the query spectrum relative to the test spectra, eliminating differences in calibration and/or the solvent dielectric.

## Usage

***Access*:** DM@PCDDB is accessible either directly via the http://pcddb.cryst.bbk.ac.uk/dichromatch.php URL, or as an option once the user has accessed the Protein Circular Dichroism Data Bank at http://pcddb.cryst.bbk.ac.uk/home.php and chooses the option (left hand panel) for DichroMatch.

### Input parameters

DM@PCDDB enables the user to either choose an existing PCDDB file or to upload their own data file for undertaking the match. If the user uploads their own file, several file formats are accepted: .pcd (that of PCDDB entry formats), .gen (a commonly used generic file produced by CDtools processing software^6^ or available as an output format of the PCDDB or all synchrotron circular dichroism instruments) or a simple 2 column format (wavelength and value) that can be created from any commercial CD instrument ASCII file, or obtained as an output format from the DichroWeb analysis server,^7^ or can be simply created from other file formats using spreadsheets. Furthermore, DM@PCDDB accepts data files expressed in either of the commonly used units for CD data (delta epsilon or mean residue ellipticity). The matching parameters used will either be default parameters, or user‐provided parameters such as the start and end wavelength regions of the spectra to be matched. This latter option enables the user to exclude data that are not deemed to be of high quality (often at the low wavelength end of a spectrum where the instrument high tension (HT) values are too high to enable accurate measurements), or simply to choose a specific region, such as the region between 218 and 230 nm which is mostly attributable to helix content. It then allows users to choose which of the comparison types to make, the maximum number of identified matches, and to define a similarity limit for the matched spectra by specifying a goodness‐of‐fit criterion (NRMSD,^8^ the normalized root mean square deviation between the input and matching spectrum) defined as:

$$\text{NRMSD}\;={\left[\frac{\sum {\left(\theta_{\text{exp}}-\theta_{\text{cal}}\right)}^{2}}{\sum {\left(\theta_{\text{exp}}\right)}^{2}}\right]}^{1/2}$$

The magnitudes of the NRMSD values will differ significantly between methods, but within any particular method, the match with lowest value for this parameter will indicate the closest fit spectrum. For the Simple Fit method specifically, the advice is given that usually an NRMSD of ≤ 0.10 indicates a strong spectral similarity.

For each of the parameters to be input, there is a help facility which can be accessed by hovering over the “?” to the right of the parameter input box. The default values of the parameters are also listed in this help facility.

### Output information

**Figure 1:**
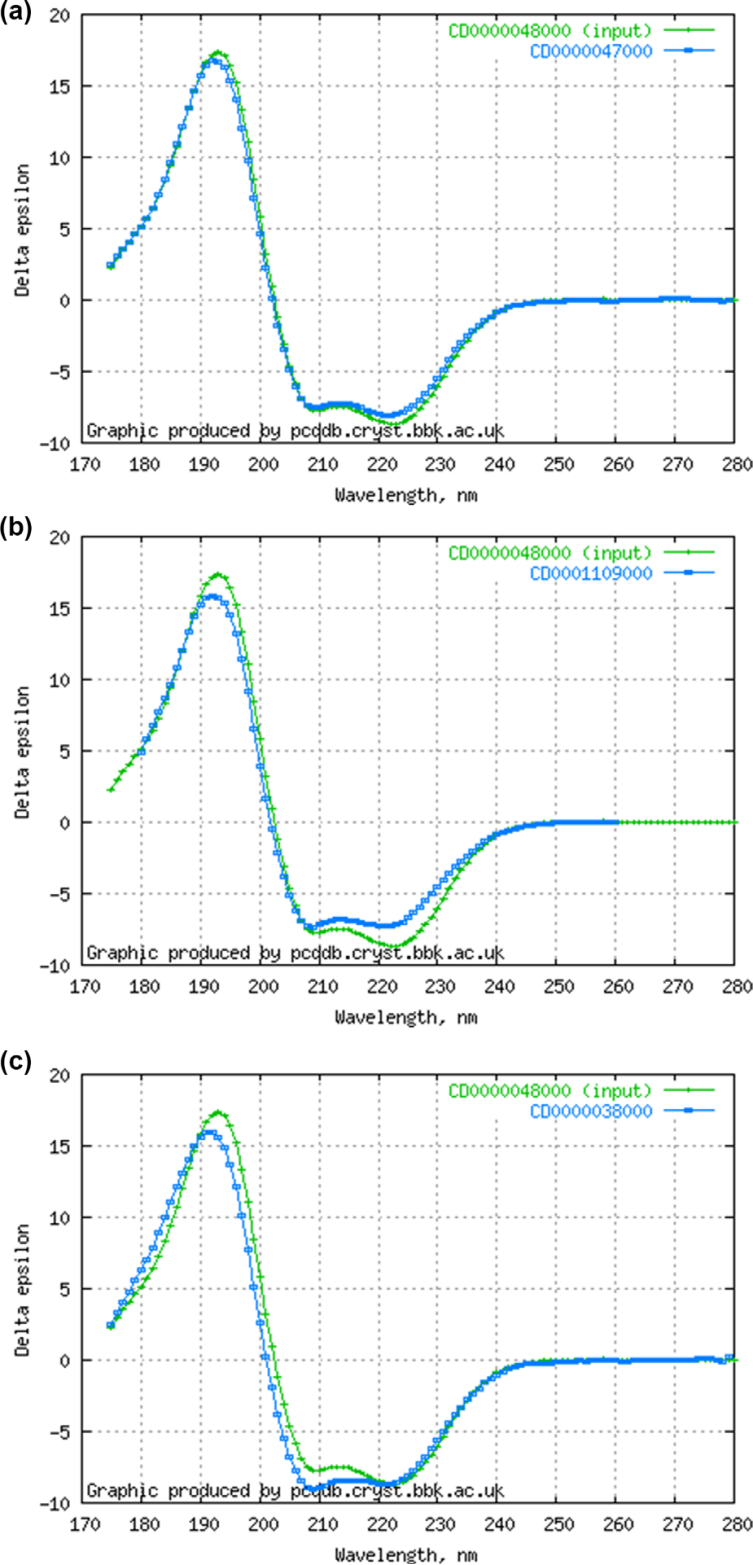
(a) Plot of DM@PCDDB output showing a close match (NRMSD = 0.069) between sperm whale myoglobin and horse myoglobin using the “simple fit” method, (b) plot showing a modest match (NRMSD = 0.135) for the same query protein with the sodium channel BH1501, and (c) plot showing a poorer match (NRMSD = 0.176) of the query protein with human serum albumin using the same method.

The output lists the query parameters used, and the spectra that meet the match criteria. For the latter, both the name of the protein (as listed in the PCDDB) and its PCDDBid are returned. The latter is listed as a clickable link to the entry so the user can easily access the information on that protein, including, if a crystal structure exists, its secondary structure. If the query protein is a PCDDB entry, it will be the first in the list of matches, with an NRMSD of 0.0. The NRMSD values for the other matched proteins are also provided. In addition, on the right hand side of the panel, the user is provided with an overlay of the spectrum (Figure 1) of the query protein with the spectra of the matched proteins, so they can visually assess the similarity themselves. For the Wavelength Shift method, the plot also includes the shifted spectrum, and above the plot, the magnitude of the shift (in nm) is given.

## Example of Usage

The spectrum of myoglobin from the sperm whale (PCDDB ID CD0000048000^3^ was tested with the Simple Fit algorithm. Not surprisingly, its top matches were with myoglobin from horse (CD0000047000) (NRMSD = 0.069) (Figure 1a). The two myoglobins are closely related proteins, based both on sequence identities (88%), and secondary structure (both are 74% helical), as well as having the same CATH^9^ class, architecture, topology, and superfamily (1.10.490.10). The next best match was to human hemoglobin (CD0004692000) (NRMSD = 0.104), which has a helix content of 69% and a sequence identity of only ∼24%, but is represented by the same CATH classification. Interestingly, the myoglobin spectrum also matches reasonably well (NRMSD <0.20) with a number of membrane protein files. These include a number of matches to the sodium channel BH1501 (CD0001109000) (NRMSD = 0.135) (Figure 1b), which is also a helical bundle (with its estimated helix content being a similar 66%); however, it has essentially no sequence identity to hemoglobin. An example of a poorer match (NRMSD = 0.176) is with human serum albumin (CD0000038000) (Figure 1c), a protein which has a similar helix content of 69%, but a negligible sequence identity (∼6%), although it shares the same CATH class and architecture levels (1.10.246.10) but not topology and superfamily.

These results indicate that the spectral matching was based on fold architecture as well as secondary structure, even if the proteins were only distantly related by sequence. This suggests that DM@PCDDB can thus be an additional, and possibly orthogonal tool, to other methods for identifying similar features in proteins.

## Conclusions

DM@PCDDB provides a simple means for comparing and identifying proteins with similar CD spectra as a potential means for finding proteins with similar secondary structures, folds or other spectral‐contributing characteristics.

## Availability

DM@PCDDB is an updated and improved version of the original DichroMatch^2^ program. It is freely accessible via the Protein Circular Dichroism Data Bank site either at http://pcddb.cryst.bbk.ac.uk/dichromatch.php or as an option at http://pcddb.cryst.bbk.ac.uk/home.php. Users should cite this paper as the reference for usage.

## Competing Interests

The authors declare no competing interests.

## References

[1] Whitmore L , Wallace BA (2008) Protein secondary structure analyses from circular dichroism spectroscopy: methods and reference databases. Biopolymers 89:392–400. 17896349 10.1002/bip.20853

[2] Klose DP , Wallace BA , Janes RW (2012) DichroMatch: A website for similarity searching of circular dichroism spectra. Nucleic Acids Res 40:W547–W552. 22638573 10.1093/nar/gks449 PMC3394267

[3] Lees JG , Miles AJ , Wien F , Wallace BA (2006) A reference database for circular dichroism spectroscopy covering fold and secondary structure space. Bioinformatics 22:1955–1962. 16787970 10.1093/bioinformatics/btl327

[4] Abdul‐Gader A , Miles AJ , Wallace BA (2011) A reference dataset for the analyses of membrane protein secondary structures and transmembrane residues using circular dichroism spectroscopy. Bioinformatics 27:1630–1636. 21505036 10.1093/bioinformatics/btr234

[5] Whitmore L , Miles AJ , Mavridis L , Janes RW , Wallace BA (2017) PCDDB: New developments at the Protein Circular Dichroism Data Bank. Nucleic Acids Res 45:D303–D307. 27613420 10.1093/nar/gkw796 PMC5210608

[6] Lees JG , Smith BR , Wien F , Miles AJ , Wallace BA (2004) CDtool ‐ an integrated software package for circular dichroism spectroscopic data processing, analysis, and archiving. Anal Biochem 332:285–289. 15325297 10.1016/j.ab.2004.06.002

[7] Whitmore L , Wallace BA (2004) DICHROWEB, An online server for protein secondary structure analyses from circular dichroism spectroscopic data. Nucleic Acids Res 32:W668–W673. 15215473 10.1093/nar/gkh371 PMC441509

[8] Mao D , Wachter E , Wallace BA (1982) Folding of the mitochondrial H+‐ATPase proteolipid channel in phospholipid vesicles. Biochemistry 21:4960–4968. 6291595 10.1021/bi00263a020

[9] Sillitoe I , Lewis TE , Cuff AL , Das S , Ashford P , Dawson NL , Furnham N , Laskowski RA , Lee D , Lees JG , Lehtinen S , Studer RA , Thornton J , Orengo CA (2015) CATH: comprehensive structural and functional annotations for genome sequences. Nucleic Acids Res 43:D376–D381. 25348408 10.1093/nar/gku947 PMC4384018

